# Impact of Social Media on Health-Related Outcomes Among Older Adults in Singapore: Qualitative Study

**DOI:** 10.2196/23826

**Published:** 2021-02-17

**Authors:** Madeline Han, Xin Yi Tan, Rachael Lee, Jeong Kyu Lee, Rathi Mahendran

**Affiliations:** 1 Department of Psychological Medicine Yong Loo Lin School of Medicine National University of Singapore Singapore Singapore; 2 Saw Swee Hock School of Public Health National University of Singapore Singapore Singapore; 3 Academic Development Department Duke-NUS Medical School Singapore Singapore

**Keywords:** aging, social media, health, qualitative research, communication, mobile phone

## Abstract

**Background:**

The worldwide spread of digitalization has led to the harnessing of technology to improve health outcomes. Paying attention to older adults’ social needs via social media is one way to promote healthy aging. Although 56% of older adults are smartphone users, little is known about their use patterns of social media.

**Objective:**

This exploratory study aims to determine the experiences of social media apps’ use among older adults in Singapore and understand their perceptions of its impact on health-related outcomes.

**Methods:**

This study used a qualitative research design with an interpretative approach. Using maximum variation purposive sampling, normal aging older adults (N=16) who were aged between 60 and 80 years and experienced in the use of internet-enabled technology were recruited from an existing community study. Semistructured, in-depth interviews were conducted. Employing a thematic analysis, interviews were transcribed verbatim and analyzed for codes inductively.

**Results:**

The following themes and subthemes were identified as key moderators of older adults’ experiences on social media apps: (1) personal attitudes: participants were encouraged to use social media due to the increased accessibility, which enabled the ease of contact, but perceptions that the quality of interactions was compromised and its associated risks reduced their use; and (2) social influences: the desire to bond with co-users and the availability of support increased use. In addition, use of social media apps was perceived to positively impact health through its ability to keep older adults cognitively engaged, improve health communication, and increase social connectedness. However, opinions remained mixed on older adults’ vulnerability to social media addiction.

**Conclusions:**

Personal and social contexts determine older adults’ social media use. This study’s findings provide practical insights into how social media can be deployed to improve health-related outcomes in older adults.

## Introduction

### Background

Technological advances provide opportunities to meet the social needs of older adults and to educate and empower them on health-related matters. Social media refers to internet-based platforms that use an electronic means of communication to enable social interactions via the consumption, generation, sharing, and exchange of ideas and content by users within their virtual communities [1]. It includes social messaging apps (eg, WhatsApp), social networking sites (SNSs, eg, Facebook), and media-sharing apps (eg, Instagram). These platforms yield benefits over traditional communication modalities, such as telephones and regular text messaging, as real-time updates and communications are not bound by geographical distances [2]. The audiovisual functionalities of social media permit the sharing of pictures, videos, and audio recordings, which trigger the realism of depicted activities and increase feelings of being *socially present* [3]. In addition, the level of interactivity offered on SNSs is positively associated with greater social bonding with family members [4].

The internet is a preferred source of health information for older internet users [5,6], rendering it a potential tool to improve health communications for older adults. Health blogs and online support groups on SNSs could serve as useful resources for disease management in older adults [7,8], psychological well-being [9], and cognitive functioning [10,11]. However, there is limited research investigating the potential negative health impact of social media among older adults.

The feasibility of social media as a health promotion tool depends on the acceptance of technology. The traditional view is that a digital divide exists [12], with older adults typically being slower than younger adults in adopting new technology. Although research suggests that functional limitations such as cognitive impairment limit capacities to adapt to new technology, older adults are more likely to adopt the technology if they are personally interested and willing to invest in the effort to learn and use the technology [13-15], possess beliefs in self-efficacy [16], and are socially motivated to engage in intergenerational communication with family members [4,13,17]. Fears of privacy breaches [13,14,17,18], a lack of confidence [15,19], and perceptions that web-based communication is trivial [18,19] are some barriers to social media use. These findings indicate that social media use largely hinges on older adults’ own attitudes and beliefs, implying an artifactual digital divide that can potentially be overcome.

### Objectives

One of the ways to access social media is the use of social media apps on smartphones. In Singapore, approximately 56% of those aged 60 years and above are smartphone users; 73% have used it to access internet-enabled platforms, including social media apps [20]. Furthermore, Singapore has actively pushed to become a *Smart Nation* with several initiatives to increase information technology literacy in seniors, including short courses to promote skills learning (eg, operating a smartphone) and becoming *social media–savvy* [21]. From a public health perspective, with the growing adoption of smartphones and Singapore’s move toward a *Smart Nation*, it appears that social media apps could be tapped on as useful platforms to promote health-related outcomes among older adults. An understanding of older adults’ perceptions of social media apps could also enable us to evaluate the utility of social media apps as health promotion tools in this age group.

However, despite the potential offered by social media apps, there seems to be scarce literature investigating older adults’ perceptions and attitudes specifically toward social media apps. Although a previous local study examined smartphone adoption and identified generally positive attitudes toward smartphone use among older adults [22], it is unclear whether smartphone use encompasses use of social media apps. Furthermore, much of the current research focuses on attitudes toward either SNSs only (eg, Facebook) [17] or technology acceptance in general [13]. Hence, this study seeks to extend the literature by (1) exploring the experiences of older adults in Singapore in their use of social media apps in enabling interactions and (2) understanding their perceptions of how social media app use influences health-related outcomes. As this was an exploratory study, no a priori hypotheses were made.

## Methods

### Study Design and Sampling

This study employed a qualitative research design with an interpretative approach. The study sample was recruited from the Community Health and Intergenerational (CHI) study of community-living older adults aged 60 years and above in Singapore [23]. The CHI study is an existing 3-year cohort study that aims to comprehensively investigate health profiles of 1000 older adults by looking at the biological, psychological, and social factors associated with the aging process [23].

The following inclusion criteria were established: (1) age between 60 and 80 years, (2) normal aging (established in neuropsychological tests in the CHI study), and (3) experience with use of internet-enabled technology (determined by smartphone ownership and ability to operate it). In addition to the above, maximum variation purposive sampling was used to ensure diversity of views and opinions. To this end, key participants from different genders, age groups, language of communication, and living arrangements were selected from the list of eligible participants who met the inclusion criteria.

### Procedure

Written informed consent was obtained from all participants. At the start of each interview, participants were asked to list the social media apps they used and indicate the frequency of use of each app (to quantify use patterns). Semistructured, in-depth interviews were conducted with an interview guide (Textbox 1), which was developed from a systematic review of factors found to influence technology adoption to support aging-in-place [24]. Participants also answered additional questions on their social media use and its impact on health.

**Interview guide (factors and examples of interview questions).**

Concerns regarding technology and benefits of technology

- “Can you give me an example of a benefit/drawback of social media?”

Need for technology

- “What made you decide to start using social media?”
- “What do you use social media for?”
- “(For low-frequency users) Why do you feel that social media is not important to you?”

Available alternatives to technology

- “What are the differences between social media apps and traditional communication platforms?”
- “If there is no social media, how do you think your communication would be like?”

Social influences

- “How do your friends/family members feel about your social media usage?”

Impact of social media use on health

- “In your opinion, do you feel that social media has any direct or indirect impact on health?”
- “Can you give me some examples of a time when you felt that social media affected your health?”

Interviews were conducted by a single trained interviewer in either English or Mandarin and were audio recorded. Participants were free to respond in colloquial speech [25]. Data analysis was conducted concurrently with the interviews, and recruitment ceased when thematic saturation was reached with 16 participants (S01 to S16). Interviews lasted approximately 1 hour and 2 minutes (SD 13.4 minutes) and were conducted between February and May 2019. Ethics approval was granted by the National University of Singapore Institutional Review Board (S-18-379).

### Data Analysis

Interviews were transcribed verbatim. To mitigate reporting bias on time spent on social media, transcripts were further examined for details of older adults’ use patterns before categorizing into low, average, and high. A thematic analysis with an inductive approach was used to identify and categorize codes using the QSR NVivo 12 software [26]. The transcripts were coded line by line. Two coders were involved in data coding and analysis. To increase inter-rater reliability, one coder coded 5 random transcripts, whereas another coded the rest. The coders then discussed the preliminary codes, subthemes, and key themes that emerged and resolved discrepancies to ensure coherence in the interpretation of the themes. Transcripts were reread, and coding ceased when further analyses did not produce new codes.

Two interviews conducted in Mandarin were translated directly to English by an independent research assistant proficient in both languages. These transcripts were cross-checked to ensure that there were no discrepancies and that nuances in communication were captured appropriately.

## Results

### Overview

A total of 16 participants participated in this study. Table 1 summarizes the sociodemographic information of the participants. All participants used multiple social media apps. Of the 16 participants, 100% (16/16) used Facebook and WhatsApp, 69% (11/16) used YouTube, 31% (5/16) used Twitter, and 25% (4/16) used Instagram. Participants were categorized into low-, average-, and high-frequency social media app users based on the time spent on social media apps per day and their use patterns. Among the 16 participants, 31% (5/16) were low-frequency users (spends <1 hour on social media apps per day and uses social media apps on the move), 44% (7/16) were average-frequency users (spends at least 1 hour on social media apps per day and uses social media apps on the move), and 25% (4/16) participants were high-frequency users (spends at least 1 hour on social media apps per day and sets aside dedicated times to use social media apps).

**Table 1:** Sociodemographic data of participants (N=16) and the distribution of social media use by sociodemographic groups.

Characteristics		Total sample (N=16), n (%)	Social media use per characteristic		
			Low, n (%)	Average, n (%)	High, n (%)
**Gender**					
	Female	8 (50)	2 (25)	3 (38)	3 (38)
	Male	8 (50)	1 (13)	4 (50)	3 (38)
**Age group (years)^a^**					
	60-69	12 (75)	4 (33)	7 (58)	1 (8)
	70-79	4 (25)	1 (25)	N/A^b^	3 (75)
**Language of communication^c^**					
	English	14 (88)	5 (36)	5 (36)	4 (29)
	Mandarin	2 (13)	N/A	2 (100)	N/A
**Housing type**					
	1-2 room public housing	3 (19)	2 (67)	N/A	1 (33)
	3, 4, or 5 room public housing	4 (25)	1 (25)	2 (50)	1 (25)
	Executive apartment or maisonette	2 (13)	1 (50)	N/A	1 (50)
	Private housing	7 (44)	1 (14)	5 (71)	1 (14)

^a^Mean age 66.19 years (SD 5.69).

^b^N/A: not applicable.

^c^Mean schooling 15 years (SD 3.41).

The findings revealed 2 main issues related to social media apps’ use among older adults. First, we found that social media apps’ use among participants was moderated by personal attitudes and social influences. Second, participants perceived social media apps’ use as both positive and negative influences on health-related outcomes.

### Social Media Use Moderators

Two major themes were found to be key moderators of participants’ social media apps’ use: (1) personal attitudes and (2) social influences. Each theme was further divided into subthemes, representing factors that determined the frequency of social media apps’ use (Table 2).

**Table 2:** Overview of themes and subthemes: older adults’ experience in their use of social media apps.

Theme	Subtheme	
	Increases use	Reduces use
Personal attitudes	Accessibility in enabling ease of contact	Compromised quality of communication Perceptions of risks
Social influences	The desire to bond Availability of support	N/A^a^

^a^N/A: not applicable.

#### Theme 1: Personal Attitudes

##### Accessibility in Enabling Ease of Contact

All 16 participants unanimously agreed that with the advent of social media, initiating contact with peers and loved ones and group communications is easier compared with the past when they only relied on traditional modes of communication, such as text messaging and phone calls:

In my parents’ time, he (father) had to make an effort to call his friends,...somebody must organize for them to meet. Whereas now with WhatsApp, it’s just a touch of the phone!

S02

As most social media apps have no cap on use and are free to use, it encouraged more liberal and frequent contact. Participants expressed an overt preference for them:

Some people call in must pay. So they tend to have the phobia there that you call me, they dare not talk long.

S13

##### Compromised Quality of Communication

Some participants (n=3), however, perceived the quality of communication over social media to be compromised. It did not afford the same kind of emotional intimacy, such as “the feeling of the other person” that face-to-face interactions provide (S16). Even traditional communication tools, such as phone calls, were perceived as “more personal” (S13).

Ironically, the ease of contact led participants to perceive web-based interactions as brief and superficial in nature:

You want to wish people, you just put a picture then “happy birthday”—there is no contact, nothing. I’d rather call the person. The sincerity is not there.

S06

Insofar as a willingness to share, social media provides unfiltered access to the personal moments of one’s lives in real time. Some participants (n=6) perceived such communication to be trivial and excessive, noting that it “takes up a lot of the person who has to look at it, but may not be interesting enough” (S16). Describing them to be “active in a very senile way” (S12), some participants (n=3) expressed their annoyance at peers who exhibited such tendencies and actively avoided their content when surfing social media:

I have this friend; she would upload all her photos on her Facebook. Full 80 photos..., what is the purpose? I wouldn’t bother to look through.

S11

##### Perceptions of Risks

As new adopters of digital technology, several participants (n=11) stated that they are vulnerable to risks, as social media permits unfettered access to others whose intentions remain unknown. Participants pointedly refrained from self-disclosure on social media and took precautions to avoid posting self-identifying details:

I always tell them not to do too much (posting of personal photos), there are people who edit and put funny things inside.

S13

Yet, while exercising caution in their posting habits and being selective in who they added to their networks, participants remained undeterred by security risks, as they “enjoy reading other people things on Facebook.” Moreover, S11 highlighted the importance of context in allaying these fears. The knowledge that Singapore is a relatively safe and crime-free country meant that she did not perceive being *open* on social media as a threat:

Privacy to me, there is nothing to fear. Singapore is safe-lah. If I were in [name of country], I wouldn’t dare to be so active.

S11

#### Theme 2: Social Influences

Two main social influences shape older adults’ experiences on social media apps: (1) the desire to bond with co-users and (2) the availability of support from their social network.

##### The Desire to Bond

All participants (n=16) cited keeping up with others as the primary motivation in their social media use. Older adults saw its adoption as an opportunity to bridge the intergenerational communication gap and to bond with younger family members:

Because I see the children using, I just want to link with my grandchildren.

S14

Even for participants who were minimally active in their use by choice, they raised a caveat that despite their aversion, they valued the access granted by these platforms to be more involved in their family’s lives:

Another motivation is like my friends say there are things that my children are doing but I don’t know. I better get on to see what they are doing.

S08

##### Availability of Support

Participants reported the availability of different sources of support as motivation to use social media. These included family members (n=5) and peers (n=6). For example, when encountering difficulties with social media, participants would enlist the help of younger members of their social networks deemed to be “more knowledgeable” (S03). One participant credited her proficiency in social media use to being “lucky to have children”; she could easily learn by simply “ask(ing) them and they will tell me and teach me” (S11). The converse view was validated by S08, who cited the lack of support from her “less than encouraging” children as a reason for her lack of presence on these apps:

My children will only say, nobody teach me how to do it. Because of that, I’m still not on it.

As with family, peer influence was important in shaping older adults’ experiences. Digitally literate peers were usually the first point of contact for participants to learn about new social media apps:

Normally is friend recommend—my group of friends say, we use this app, so I will use.

S01

Participants exhibited greater willingness to use apps introduced by these peers and judged their recommendations as safe. As S01 explained, “Should be they (peers) won’t harm you-lah.” Often, these peers were the *go-to* people when participants needed help with the apps, if their children were too *impatient* or *fast* for them to follow. This support was emphasized by S07, who noted that although his own family members were “quite discouraging” when he attempted to grasp the use of Facebook, his colleagues rendered assistance to him and helped to “start (an account) for him.”

Novice peers were equally important in facilitating social media use. S13 explained that the support she received from her peers while learning together in a group setting had a positive effect on her emotional well-being:

It helps me in that I’m not the only one that my grandchildren are not helping, I always have to go to friends, I’m not the only one. You don’t feel neglected.

S13

### Perceived Influence on Health-Related Outcomes

Participants perceived social media apps’ use as both a health benefit and a health threat. Specifically, social media use was perceived to bring about positive outcomes, as it keeps older adults cognitively engaged, improves health communication, and increases social connectedness. Opinions remained mixed on whether social media addiction was perceived as a potential health threat among older adults.

#### Theme 1: Cognitive Engagement

Social media use allowed older adults to remain cognitively engaged. Some participants noted that social media use provided an avenue for them to acquire new skills and keep cognitively engaged postretirement (n=11), whereas others noted that social media use helped them to maintain their language skills (n=2).

Although it was daunting to grasp new knowledge and learn to use multiple functions, participants reported being motivated to learn more and sign up for courses. They viewed these learning opportunities as helping them stay “alert” (S13), such that “they won’t go senile and get dementia” (S05). Older adults perceive user-generated content on social media apps as a means to compensate for the lack of cognitive engagement as they transit into retirement. For instance, S03 described the experience of posting photos on Instagram as a way to “see your creativity” with his photography skills. Beyond developing technical expertise, the spread of fake news and misinformation on social media hones older adults’ abilities to judge the credibility of its content:

You know how to differentiate whether good or bad. Not all news give(s) you the correct information, so you're more sharp of that.

S03

Other participants cited concerns over their linguistic abilities post retirement and viewed social media posting as a way to “sharpen the brain,” such that “the spelling, the use of words will not be dropped off from your memory” with old age (S08).

S12 credited social media use to provide an outlet for him to articulate his opinions on news topics, which allowed him to practice his writing and thinking skills:

Being able to even write things every day. How you construct a sentence, how you convey an idea...it should help with your cognitive ability.

However, S12 highlighted that those who “are the type who just forward” may not be cognitively engaged if thinking is not involved.

All participants (n=16) acknowledged that they used social media as an alternative news source. As S08 explained, social media is an additional channel to receive up-to-date news, more than what she would have been exposed to via traditional media:

It does get me into reading more than what I’m already reading or watching.

#### Theme 2: Health Communication

Participants agreed that social media increased their accessibility to a wealth of health knowledge. This ranged from common health information on nutrition (n=4), complementary and alternative medicine (n=6), or advice that advocates engagement in behavior (eg, special exercise techniques) to prevent age-related decline (n=2):

They give you like what (health food) to eat more, what fruits are good, bad..., like “oh eat more ginger.”

S01

Participants particularly appreciated the access to individuals who they saw as unofficial health experts and perceived health information from these sources to be more valuable than those from their own clinicians. As S14 remarked:

All doctor(s) want you to pay money one.

However, some participants (n=2) acknowledged that with the ease of posting on social media, information circulating on social media is largely unfiltered and can be *contradictory.* The experience of trying to discern the legitimate sources of health information was *confusing* for some, and they explained the following:

For every information they share, the video says it’s good, there’s another one that says it’s not good.

S12

A few participants (n=5) opined that as older adults, they are cognizant of this and are “mature enough not to fall into the trap.” (S02). Some (n=3) attempted to cross-check with other news sources, such as other media platforms, to establish its veracity:

Sometimes I go YouTube to check whether true or not.

S01

One participant regarded traditional news media as a source of scientific authority and used it as a heuristic in deciding on what they read was believable:

When I see on TV the news is correct. But other news like Panadol, they say don’t take because can cause what, I won’t forward. Because I didn’t see in the news.

S13

#### Theme 3: Social Connectedness

S02 opined that in the past when the quality of information and communications technology (ICT) was weaker, opportunities to socialize tended to diminish with age because it was difficult to sustain interactions on a regular basis. In contrast, with social media, the ease of contact meant that its frequency will consequently increase, enabling tangible opportunities for interactions and allowing relationships to withstand time:

Look at my mother, she retired not into social media, her world became smaller and smaller. I think when I reach 80, I would have a wider base because of social media.

S02

Moreover, older adults appreciated that they were able to harness the power of social media to improve or expand existing social networks (n=9). A core feature of SNSs is that they aid the discovery of lost contacts, and few participants (n=7) reported becoming reacquainted with individuals they had previously lost touch with. Recounting an episode where she reconnected with some ex-classmates from an old photo that was posted on a Facebook alumni page, S01 noted:

I feel good. It’s just to get to know my own friends, some maybe pass away already, or they have been doing well. I just want to know their well-being only.

Importantly, this initial contact facilitates offline interactions as well. S11 related:

...through Facebook, I got connected to my school friends (from [name of country]). We managed to meet up and have a reunion!

However, S06 felt that the increased reliance on web-mediated interactions worsened the quality of relationships in real life:

People get so distant. Social media doesn’t help you to be a social person, it gets you more onto a machine.

#### Theme 4: Addiction

Participants were divided on whether social media addiction was a consequence of its use. One participant reasoned that as older adults grew up in an era where the use of technology was less integral to everyday life, older adults would be less dependent on social media and did not perceive addiction to be a problem for their age group:

They don’t have this habit from young. They picked up the knowledge only in the later stage. So it is something extra, rather than essential.

S09

Others (n=4) felt that due to their life experiences, older adults are more likely to be more “disciplined” and “aware” of the frequency of use of their smartphones as compared with youths (S02). As such, they are better able to manage the time spent on social media more effectively.

Nonetheless, with the wide variety of social media apps and the wealth of information and functions each offer, some participants (n=4) noted that new users could become deeply captivated by social media to the extent that they may be inclined to overuse it. As S05 explained:

Those who start to explore, go deep into it.

Other participants (n=4) reasoned that with postretirement, the amount of leisure time at older adults’ disposal significantly increased, allowing them to spend more time on other activities. These participants found themselves vulnerable to the allure of social media:

At one stage, I was doing this too much. Older people, especially when you are free you see. So, you tend to answer, do this.

S13

## Discussion

### Principal Findings

This study extends prior work by investigating older adults’ perceptions of social media in Singapore, with a particular focus on social media apps’ use. Consistent with existing literature, older adults’ social media use depends on personal and social contexts [13]. Despite perceived security threats, personal contexts such as personal attitudes and accessibility to communications encouraged social media use. In addition, older adults reported the desire to bond and support from others as motivations for using social media.

Of particular interest to our study are the health-related outcomes brought about by social media use. The remainder of this discussion, thus, focuses on these outcomes and the accompanying consequences. The utility of social media in health promotion for older adults, particularly in the area of healthy aging, was also evaluated.

### Social Media and Health-Related Outcomes

#### Cognitive Engagement

In this study, the role of cognition in influencing social media use was apparent—social media provided an avenue for older adults to remain cognitively engaged, while reducing their fears of future cognitive deficits. Other studies have also found that the acquisition of technical skills through social media use was associated with better neuropsychological test scores among novice older users [10,11]. Some have posited that an association exists between sociality and cognition, such that interacting with others improves cognitive functioning due to the multiple inferential processes involved [27,28]. The increased frequency of contact enabled by social media could be a possible mediating factor between sociality and cognition.

#### Improved Health Communication

Social media can be used for health communication purposes owing to its wide reach and availability of different sources of information. Interestingly, although one could obtain health information from formal sources such as physicians and health institutions, older adults appreciated the ability to acquire health information from informal sources via social media, such as individuals they deem as unofficial health experts. This highlights the importance of social relations in health communication—older adults are more likely to seek and obtain health information from individuals with whom they are socially connected (including both web-based and offline relations), compared with those who they have no connections with (eg, formal health sources). With the ability to connect to informal health sources via multiple channels, health communication is enhanced with social media use.

Although there is increased access to health information, those provided by informal sources may not be scientifically accurate, rendering the evaluation of their legitimacy challenging [29]. In addition, the sensationalism of health news [30] and the brevity of social media news increase the likelihood of readers receiving false information from them. Older adults are often vulnerable to web-based scams, and those aged above 65 years have been found to be 7 times as likely to share fake news than younger users [31]. Although older adults are aware of the need to establish the veracity of news encountered on social media, their strategies in doing so may be questionable. This suggests the need for further interventions to foster digital literacy skills among older adults to help them better evaluate health news. Different strategies, such as face-to-face classes and blended learning workshops [32,33], could be adopted to teach digital literacy skills to older adults.

#### Increased Social Contact and Connectedness

This study indicates that the accessibility of social media in enabling communication and its consequent increase in social contact present a means to preserve dispersed social relationships as older adults transit into retirement. Additional social contact could enrich the quality of social relationships and enhance emotional well-being, as evidenced by studies that found that SNS use was associated with perceptions of feeling valued and supported through their social networks and deriving greater satisfaction with life [34,35].

However, an increase in social contact may not necessarily translate to social connectedness. This is dependent on the motivations behind social media use: whether engagement with others is motivated by the *reward* of prospective social ties or by the desire to avoid the negative feelings associated with social isolation [36]. For the former, social media strengthens relationships and increases social connectedness. For the latter, time spent on social media is seen as time spent away from real-life interactions, which are perceived to generate greater value [36]. Moreover, if efforts made to initiate web-based contact with other co-users are not reciprocated, the effectiveness of social media in improving social connectivity may be limited [37].

The varying views of the value of web-based social interactions suggest that social media supplements, rather than replaces the need for offline, face-to-face interactions. As can be seen from our findings, those with positive social media experiences emphasized the ease of facilitating offline interactions (eg, reunions with acquaintances or meet-ups with their social circles) as a key benefit of social media. Thus, for social media use to be meaningful, web-based interactions should be positive; rewarding; and present opportunities for fostering additional offline, face-to-face interactions. Beyond encouraging social media use, future intervention programs should also aim to provide more opportunities for older adults to connect with others through offline interactions.

#### Risk of Addiction

Social media addiction, characterized by its excessive use and lack of self-control over use habits, is a potential health concern. Although little has been elucidated for older adults, a Norwegian study reported that approximately 1% of those aged 60 to 74 years were found to be at risk for internet addiction [38]. The prevalence of internet and social media addiction is expected to rise with the rapid spread of digitalization today. Considering this, the findings of this study that some older adults perceive themselves as invulnerable to social media addiction and the possibility of excessive social media use due to an increase in leisure time postretirement warrant concerns.

### Social Media and Healthy Aging

#### Potential in Reducing Social Isolation and Loneliness

Older adults are often at a high risk of social isolation, as their social networks begin to shrink. In Singapore, 73.4% of older adults are at least at a moderate risk of social isolation [39]. Furthermore, social isolation is associated with poorer health and cognitive outcomes such as increased mortality and multimorbidity risks [40] and increased risk of developing dementia [41]. Thus, social isolation is a public health concern.

Older adults with physical disabilities are at an even higher risk of social isolation. Nonambulant individuals are physically limited in the range of social activities they can engage in [42], whereas those with hearing disorders may deliberately choose to socially withdraw due to potential difficulties in verbal communication [43]. For these individuals, social media may be an effective tool to facilitate social interactions and contact, as they will not be bounded by physical spaces or abilities. Previous studies have cited the successful use of internet training and access in reducing social isolation [44-46], suggesting the possibility of tapping on digital technologies and platforms for potential interventions. Future research could explore how social media could be used to ameliorate social isolation among older adults, especially those with physical disabilities.

Although often conflated, social isolation and loneliness are 2 different states. The former is a quantitative measure of social network sizes, whereas the latter is the subjective feeling of being socially isolated. In Singapore, even among older adults deemed to be at low risk of social isolation, 44.7% were *sometimes lonely* or *mostly lonely* [39], highlighting the different social needs of these 2 groups. At present, evidence supporting the effectiveness of social media in reducing loneliness remains mixed [47,48]. Furthermore, the greater visibility into the lives of their contacts afforded by the increased access on social media may abet upward social comparison and intensify feelings of loneliness [49]. Future research could be directed toward elucidating how lonely older adults perceive social media use, to determine its utility in meeting their social needs.

#### Support for Learning

It is crucial to ensure a conducive learning environment (with instrumental support) for older adults. The formation of peer learning communities could prove to be beneficial, where social media literate older adults could be engaged as volunteers to support novice users in their learning on using social media. The utility of peer learning in seniors is well documented [50-52]. Specifically, in the area of ICT, even the most proficient seniors would have encountered similar experiences in their initial forays into social media and would thus be better positioned to advise new users on the knowledge of social media apps than the younger generation [53]. Venues such as senior activity centers could be tapped upon to facilitate these group sessions.

#### Role in Successful Aging

According to Rowe and Kahn [54], successful aging comprises 3 components: maintenance of physical and cognitive functioning, delayed onset of diseases and disability, and continued engagement in social relations and meaningful activities. Although cognitive and physiological deficits experienced in older adulthood are results of age and genetic predispositions, modifiable extrinsic factors such as lifestyle habits play a part as well. Social media use could, therefore, act as modifiable factors and potentially promote successful aging by providing older adults an avenue for cognitive engagement, while facilitating meaningful web-based and offline social interactions at the same time [54]. Moreover, as older adults value the importance of contributing back to society [55], the aforementioned recommendation allows older adults to remain engaged in meaningful activities when they volunteer to assist their peers on social media navigation.

### Strengths and Limitations

One of the strengths of this study was the strong rapport the interviewer had with all participants. Consequently, participants were comfortable throughout the interviews, were forthcoming about their experiences, and did not hesitate to share their personal struggles with social media apps. This ensured the credibility of the data. Having a second coder outside of the study team also enhanced the confirmability of the findings. The use of maximum variation purposive sampling provided diverse views. Older adults were well represented across gender, age groups, living arrangements, and language of communication. Importantly, the study included non-novice users whose social media use was low. This was in contrast with existing qualitative research on ICT use, where they predominantly focused on novice users and the barriers faced toward technology adoption [19,56,57]. Our study, thus, allowed for a wider consideration of barriers across different user groups. Barriers faced by non-novice users are likely to be different from novice users, and both groups should not be conflated together.

The findings of our study may have limited transferability due to the inherent characteristics of the sample. Participants in this sample are more highly educated, having received 15 years of education on average. This demographic profile was to be expected, as participants had relatively high educational attainment in the original CHI study. The findings of this study should be interpreted in consideration of this limitation. Furthermore, using smartphone ownership as an inclusion criterion could limit this sample to only affluent participants, as older adults tend to view smartphone ownership as a status symbol [22]. Although this possibility was mitigated with the inclusion of participants residing in 1-and 2-room housing flats, further research could be conducted specifically on older adults with lower income and education levels.

### Conclusions

The study examined the social media experiences of older adults in Singapore as well as their perceptions of social media in promoting health-related outcomes. Our findings showed that the decision to use social media is dependent on personal and social contexts, and the various socialization experiences suggest that social media benefits do not apply to everyone. Nonetheless, our findings highlight multiple health benefits that could be achieved with social media, such as cognitive engagement, improved health communications, and increase in social connectedness. Health care professionals, researchers, or nonprofit organizations interested in delivering health-related information could look into using social media as a potential psychosocial intervention for older adults. Furthermore, for older adults who express keen interest in learning to use social media, every effort should be directed toward providing the necessary infrastructure to navigate the pitfalls of social media effectively.

## Abbreviations

CHI: Community Health and Intergenerational
ICT: information and communications technology
SNS: social networking site
